# Pensions and the Medical Profession

**Published:** 1907-12-07

**Authors:** Thos. W. Banks

**Affiliations:** Mervyn, Lanark


### Editor's Letter-Box.

### PENSIONS AND THE MEDICAL PROFESSION.

Sir,—The idea of this accident and illness insurance is a good one, and we hope that it will be much taken advantage of. Might I suggest another great want in the medical profession?—namely, the raising of a fund by yearly subscription whereby a pension may be gained by the doctor in his old age or, if the doctor dies in harness, a yearly allowance to be handed to the widow.

How often do we read and hear of a medical man dying and leaving his wife almost bare—subscriptions among medical friends and others being raised for the purpose of giving her another start in life. All this through no fault of the deceased.

Medical men may work hard all day and get very little for it. Accounts sent out will not be paid, while accounts coming in must be paid. Then the outgoing expenses—household, educational, horses, motors, drivers, etc.—all tell very heavily, especially on the country men, who have long distances to go and get practically nothing for it. They have a fund such as this in the ministry, and I think also in law. Why not in the medical?

This might be discussed and, if possible, acted on.

I am, yours very truly,

Thos. W. Banks.

Mervyn, Lanark, November 27, 1907.

